# Zebrafish models of cerebrovascular disease

**DOI:** 10.1038/jcbfm.2014.27

**Published:** 2014-02-12

**Authors:** Brian P Walcott, Randall T Peterson

**Affiliations:** 1 Department of Neurosurgery, Massachusetts General Hospital and Harvard Medical School, Boston, Massachusetts, USA; 2 Cardiovascular Research Center, Massachusetts General Hospital and Harvard Medical School, Charlestown, Massachusetts, USA; 3 Broad Institute, Cambridge, Massachusetts, USA

**Keywords:** aneurysm, arteriovenous malformation, cavernous malformation, moyamoya, stroke, zebrafish

## Abstract

Perturbations in cerebral blood flow and abnormalities in blood vessel structure are the hallmarks of cerebrovascular disease. While there are many genetic and environmental factors that affect these entities through a heterogeneous group of disease processes, the ultimate final pathologic insult in humans is defined as a stroke, or damage to brain parenchyma. In the case of ischemic stroke, blood fails to reach its target destination whereas in hemorrhagic stroke, extravasation of blood occurs outside of the blood vessel lumen, resulting in direct damage to brain parenchyma. As these acute events can be neurologically devastating, if not fatal, development of novel therapeutics are urgently needed. The zebrafish (*Danio rerio*) is an attractive model for the study of cerebrovascular disease because of its morphological and physiological similarity to human cerebral vasculature, its ability to be genetically manipulated, and its fecundity allowing for large-scale, phenotype-based screens.

## Zebrafish characteristics

### Vasculature

Drug discovery involves a stepwise series of processes that typically begin with biochemical and cellular assays to screen for agents of potential value, which are later validated in animal models, and ultimately in human subjects.^1^ The process is costly, resource intensive, and time consuming. The use of zebrafish (*Danio rerio*) models in this process has been used effectively to identify new drugs, discover new indications for already FDA-approved drugs, and to better understand the mechanisms for various human diseases.^2, 3^ The advantages of using zebrafish to model human diseases are numerous, and relate to their ability to study all developmental stages, coupled with a scale that is not possible in other vertebrate systems.^2, 4^ For cerebrovascular disease in particular, the zebrafish offers the unique advantage of their embryo stage being optically transparent, making it possible to study the functional and morphological changes in cerebral blood vessels in a living organism. The study of cerebral vasculature can be further highlighted with the use of transgenic zebrafish, such as Tg(flk1:GFP) that express green florescent protein in their endothelium and can be readily visualized with the use of epifluorescence microscopy.^5^ While fluorescent vasculature is not a direct measurement of cerebral blood flow, it does represent an *in vivo* means to assess vascular structures with a high level of resolution, capable of rapid phenotyping of thousands of individual subjects. Other methods of physiologic assessment include microangiography^6, 7^ and laser-scanning velocimetry^8^, which can be used to further characterize the qualitative and quantitative changes in cerebral blood flow. Furthermore, zebrafish embryos are able to undergo live imaging^9, 10^, allowing for real-time visualization of angiogenesis and vasculogenesis. In addition, the dimensions of blood vessels can be measured in any axis following fixation in resin with an extremely high degree of accuracy.^11^ These techniques allow for spatial and temporal resolution of alterations in hemodynamics and blood vessel structure, which are useful tools in the study of cerebrovascular disease.

### Genetics

For zebrafish models of disease to be pertinent to the pathophysiology of humans, they must share genetic underpinnings, not just merely a common phenotype. Although the zebrafish appears as a relatively simple organism, comparison of the two genomes has demonstrated a high degree of conservation in genes implicated in processes ranging from oncogenesis to angiogenesis.^12, 13, 14^ There is also a high degree of conservation between humans and zebrafish with respect to drug responses, indicative of a high degree of amino-acid sequence identified at protein-active sites where many drugs bind.^15^ Although the genetic sequence of zebrafish is highly conserved with humans, it is more readily manipulated for the purposes of experimentation. Several tools exist in the armamentarium of the zebrafish biologist that can be used to dissect pathophysiologic pathways. Broadly speaking, the use of ‘forward' genetic screens can be used where chemicals with both known and unknown functions, along with other mutagens, can be administered to zebrafish and their phenotype can be characterized.^16^ Conversely, ‘reverse' genetic screens can be performed where the gene of interest is manipulated precisely with one of the several methods and then the phenotype is observed. Examples of this include morpholino oligonucleotide knockdown^17, 18^, transcription activator-like effector nucleases^19, 20^, zinc-finger nucleases^21, 22^, and the clustered, regularly interspaced, short palindromic repeats (CRISPR)—CRISPR-associated (Cas) systems^23^, each of which can induce targeted genetic modifications in zebrafish embryos.

### Phenotype Screening

The size of the zebrafish facilitates a large experimental scale size that is not possible with other vertebrates. Adult zebrafish pairs can generate up to 300 embryos at each mating, allowing for experiments with thousands of organisms at a time. Because the zebrafish embryo is much smaller than 1 mm in diameter, experiments can be performed in 96 or 384-well plates. Most small molecules readily diffuse into zebrafish, requiring only a dilution of the drug into the fish water to effectively ‘treat' organisms. Tens of thousands of known and unknown compounds are commercially available, including libraries specifically generated with FDA-approved compounds.^2^ The zebrafish's microscopic size, high fecundity, and ease of drug administration, coupled with ease of phenotype recognition, make it well suited for high-throughput screening.^24^

## Zebrafish stroke models

### Cerebral Amyloid Angiopathy

Cerebral amyloid angiopathy is a cause of potentially fatal lobar intracranial hemorrhage, particularly in the elderly.^25^ Histologically, the diagnosis is characterized by deposition of amyloid peptides around brain vessels, resulting in fibrinoid necrosis, microaneurysm development, and ultimately, vascular rupture.^26^ Effective therapeutics do not currently exist that have been shown to alter the natural history of the disease and the exact pathogenesis of the condition remains elusive. One of the leading hypothesis underlying cerebral amyloid angiopathy-related hemorrhage is that beta amyloid deposition leads to endothelial dysfunction (early senescence).^27^ Emerging work in a zebrafish model has been used to explore the pathogenic mechanisms of the disease.^28^ In these experiments, beta amyloid peptide was administered via diffusion in fish water and a senescence phenotype was assessed by measuring beta-galactosidase activity and the cyclin-dependent kinase inhibitor p21 expression (*in situ* hybridization in whole-mount zebrafish embryos). This work demonstrated that amyloid deposition has activity related to the senescence of the endothelium, producing progressive alterations of microvessel morphology and function. Importantly, it also highlights the utility of the zebrafish model in the investigation of this disease. Future studies are anticipated to further elucidate the effects of beta amyloid administration in the endothelium. Given that small peptides can diffuse into zebrafish, small molecule screens to identify novel therapeutic targets may be indicated in cerebral amyloid angiopathy, Alzheimer disease, and other disorders of amyloid deposition.

### Cerebral Arteriovenous Malformation

Arteriovenous malformations (AVMs) of the brain are vascular anomalies of children and adults who carry a high risk of hemorrhage, about 2% to 4% per year over the patient's lifetime.^29^ The primary pathological phenotypic character of AVMs is a direct communication between arteries and veins without an intervening capillary bed. Treatment of these lesions, either with surgery, endovascular embolization, or external beam radiation (stereotactic radiosurgery), carries significant risks, especially when lesions are large or located in eloquent brain areas. No specific medical therapies currently exist and their pathogenesis is incompletely understood. Despite a robust understanding of vasculogenesis and angiogenesis^30, 31, 32, 33, 34, 35, 36, 37, 38, 39^, the mechanisms behind the formation of discrete AVMs are not well known. It is thought that abnormalities in blood vessel formation and segregation during embryonic development are thought to be responsible, although *de novo* and recurrent lesions have been seen in adult life and dysfunctional angiogenic processes have also been implicated.^40, 41, 42^

Arteriovenous malformations occur sporadically or much more rarely, in the context of a hereditary syndrome. One such syndrome in humans, hereditary hemorrhagic telangiectasia type 2 (OMIM phenotype ID 600376), is caused by a mutation in the gene encoding activin receptor-like kinase 1, a type 1 transforming growth factor beta receptor in the BMP signaling pathway.^43^ This known mutation provides an opportunity to study the pathogenic mechanisms of AVMs in vertebrate models.^44, 45^ Another syndromic form of cerebral AVM development, such as CM-AVM (OMIM phenotype ID 608354), is caused by a mutation in *RASA1*,^46, 47^ a gene that has been fully sequenced in the zebrafish. Even though only a small percentage of AVMs are thought to be related to these different Mendelian patterns of inheritance,^48, 49, 50^ evidence does exist that that single nucleotide polymorphisms in genes such as *alk1* occur in many sporadic occurring AVMs.^51, 52^ Therefore, further dissection of mechanistic pathways leading to AVM development may be applicable to AVMs that occur in the sporadic setting, as well as in cases of Mendelian inheritance.

**Figure 1:**
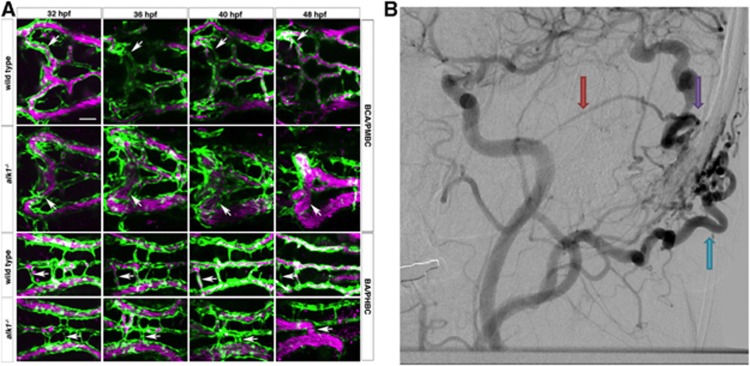
Phenotype comparison of zebrafish and human arteriovenous malformations (AVM). (**A**) In wild-type embryos (row 1), transient connections between the basal communicating artery (BCA) and primordial midbrain channel (PMBC) carry blood at 32 hpf but regress by 48 hpf (white arrows). In *alk1* mutants (row two), one or both of these bilateral connections may be retained, forming an abnormal BCA–to–PMBC arteriovenous connection (white arrows). More posteriorly, lumenized connections drain the basilar artery (BA) to the primordial hindbrain channel (PHBC) in wild-type embryos at early times, but almost all regress by 48 hpf (row 3, white arrows). In *alk1* mutants, one or more of these connections may be retained, forming a BA–to–PHBC AVM (row 4, arrows). This model resembles the human condition, seen in a digital subtraction cerebral angiogram. (**B**) In human AVMs, arterial branches (red arrows) connect directly to the venous circulation (blue arrows) through a high-flow fistula (purple arrow). One theory for AVM development is that they represent the abnormal persistence of normal transient developmental connections. Scale bars, 50 *μ*m. Zebrafish images are two-dimensional confocal projections of *Tg(kdrl:GFP)*^*la116*^*; Tg(gata1:dsRed)*^*sd2*^ embryos, dorsal views, anterior leftwards. Endothelial cells are green; erythrocytes are magenta. Human digital subtraction angiogram is a lateral projection carotid artery injection in the late arterial phase. (Figure and legend modified from Corti P *et al.*^44^ Distributed under the terms of the Creative Commons Attribution (CC-BY) License).

Several mammalian models of cerebral AVMs exist, but are limited in their applicability for drug discovery. For example, cerebral AVMs have been created in swine, but this method requires a surgical intervention to generate a lesion that mimics the human condition.^53^ Other animal models, including mice, have been used, although they require angiogenic stimulation with vascular endothelial growth factor, in addition to genetic manipulation to generate lesions.^54, 55, 56^ In comparison, zebrafish are an attractive model in that their endothelium is visualized easily with fluorescent proteins and their entire cranial circulation can be observed *in vivo*. By manipulating gene expression, AVMs can be generated in the cranial circulation of zebrafish, recapitulating the human disease with a high level of fidelity.^44, 45^ (Figure 1) Beyond the appearance of the abnormal blood vessels, zebrafish models of cerebral AVM also demonstrate systemic manifestations of the accompanying pathophysiologic hemodynamic response that is seen in humans, such as in high output cardiac failure in the pediatric population.^46, 57, 58^ The recent announcement from the National Institute of Neurological Disorders and Stroke halting enrollment in the ARUBA trial because of the procedural risk associated with any form of interventional treatment emphasizes the urgent need to find effective medical therapies for these lesions.^59^ Zebrafish models show promise in not only accelerating the discovery of pathogenic mechanisms, but also in the discovery of effective, targeted therapeutics through high-throughput screening.

### Cerebral Aneurysm

Aneurysms are lesions of the cerebral vasculature that have a typical phenotypic characteristic of an outpouching of a blood vessel wall as a result of an inherent weakness. As aneurysms enlarge, their propensity for catastrophic rupture increases.^60^ Treatment of these lesions is optimally performed prior to an hemorrhagic event, either with microsurgical obliteration or endovascular techniques.^61^ Their pathogenesis is generally thought to result from the interaction between genetic and epigenetic factors (such as cigarette smoking and hypertension).^62, 63^ Evidence for the genetic aspects of aneurysm pathogenesis is well established from powerful population based studies, such as the familial intracranial aneurysm study.^64^ In addition, informative studies, known as genome-wide linkage studies, have been performed on rare families that are affected with aneurysms.^65, 66^ What is known from these studies is that there are many genetic susceptibilities implicated in the development of familial aneurysms.^67^ Furthermore, cerebral aneurysms can occur in the setting of autosomal dominant polycystic kidney disease (OMIM phenotype ID 601313), a hereditary condition caused by a mutation in the *PKD1* gene. This syndrome has effectively been modeled in the zebrafish, where knockdown of PKD1 orthologs resulted in a distinct phenotype, related to deficiencies in extracellular matrix integrity.^68^

The understanding of aneurysm development is best examined experimentally where *in vivo* imaging and genetic analysis can be performed. Multiple types of organisms have been utilized in the study of aneurysms, including the rabbit^69, 70^, the mouse^71, 72^ and the dog.^73^ While the size of these organisms allows for detailed physiologic study, it also inherently prohibits their use in high-throughput-type experiments. The embryonic zebrafish is an ideal organism for the study of aneurysmal disease. It is completely transparent allowing for visualization of its cranial vasculature, is readily genetically modified, and has a genetic homology that is strikingly similar to humans.^12^ Angiography is readily performed allowing for determination of hemodynamic variables that are key to aneurysm pathogenesis, such as blood flow rate and wall shear stress.^74, 75^ In this way, the zebrafish approximates the human condition and allows for its manipulation in unparalleled ways.

### Cerebral Cavernous Malformations

Cerebral cavernous malformations, also known as cavernous angiomas or cavernomas, are one of the few causes of stroke known to reliably be caused by one of at least three genetic mutations.^76^ Histologically, they consist of enlarged capillary cavities (low flow, low pressure) without any intervening brain parenchyma. These lesions can result in seizure and/or hemorrhage, and therefore, the treatment is typically recommended for when they are symptomatic.^77, 78^ Currently, no therapeutics exist outside the realm surgery^79, 80^ or focused external beam radiation (stereotactic radiosurgery).^81, 82, 83^ Treatment becomes challenging, carries significant risk, or can even be considered impossible for lesions situated deep within critical brain structures. In addition, lesion multiplicity also complicates treatment suitability and is more commonly seen with familial cases.^84^

While both Mendelian inheritance and sporadic cases exist, mutations in *CCM1*, *CCM2*, or *CCM3* can be identified the majority of these lesions.^85, 86, 87^ A growing understanding of the function of their gene products has allowed for progress toward elucidating the basic mechanisms of disease pathogenesis.^88, 89, 90, 91, 92^ However, a more complete understanding of the biochemical and cellular processes that lead to the disease phenotype are necessary, and require the context of an *in vivo* assay. The zebrafish continues to be used as a model organism given its optical transparency of the embryonic stage and its ability to be genetically manipulated.

**Figure 2:**
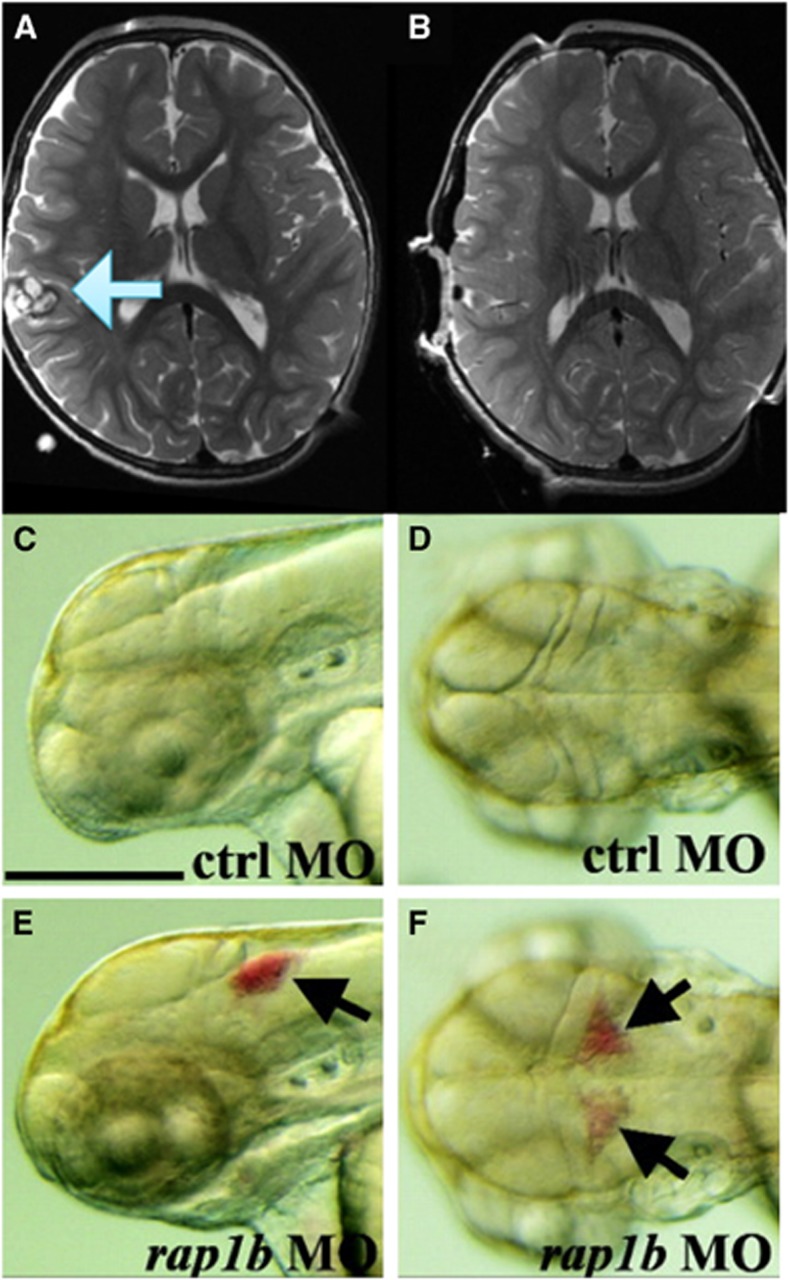
Phenotype comparison of zebrafish and cerebral cavernous malformation. In an magnetic resonance imaging of a human (**A**), a cerebral cavernous malformation is apparent in the right frontal lobe (blue arrow). These lesions can result in catastrophic hemorrhage and/or seizure activity. Treatment with surgery is effective, and complete resection can be achieved (**B**) if lesions are in accessible areas. When lesions are deep or multiple, surgical treatment may not be indicated, underscoring the need to develop novel therapeutics. To understand the molecular mechanisms in the CCM pathway, zebrafish models of the disease have been generated using morpholino technology. Compared with control organisms (**C** and **D**), morphant knockdowns of *rap1b* (**E** and **F**), a gene that encodes a Ras GTPase effector protein for CCM1/Krit1, demonstrate disrupted endothelial junctions, resulting in intracranial hemorrhage (black arrows), similar to human lesions. Bars, 250 *μ*M. (Figure modified from Gore A V *et al.*^95^ Distributed under the terms of the Creative Commons Attribution (CC-BY) License).

The zebrafish exome shares a striking similarity with humans, and the orthologs of the three genes (*CCM1, CCM2,* and *CCM3*) responsible for cavernous malformations have been identified (*santa, valentine*, and *pcdc10,* respectively).^93, 94, 95^ These mutations can be introduced in zebrafish either through genome editing or with the use of morpholino knockdown technology. Loss of these gene products results in impaired cardiovascular development, specifically a characteristic dilated heart phenotype.^96^ In addition, zebrafish with loss of function in these genes also develop a vascular phenotype, in addition to their cardiac developmental abnormalities. Looking at the cerebral vasculature, the zebrafish develop thin-walled vessels that are prone to hemorrhage, reminiscent of what is seen in pathologic human lesions.^95, 97^ (Figure 2) The experience with CCM modeling in zebrafish is one of the leading examples of how cerebrovascular disease can be studied in animal models.

### Moyamoya Disease

Moyamoya disease is a life-threatening cerebrovascular disease that predisposes patients to both ischemic and hemorrhagic stroke.^98^ The key angiographic feature that defines the condition is progressive stenosis of the intracranial internal carotid arteries and their branches. The classic configuration of their intracranial circulation is described as a ‘puff of smoke', with network of abnormally dilated collateral vessels that attempt to compensate for the lack of blood flow through the normal conduits in the circle of Willis. A variety of direct and indirect surgical revascularization procedures are used to treat the condition, each with varying rates of success.^99, 100, 101^ No pharmacologic therapy has been shown to alter the natural history of the disease. Although several susceptibility loci have been identified^102, 103^, the pathogenesis of the condition remains elusive.

Zebrafish have proven to be valuable tools in this condition by allowing further investigation of gene function. Following genome-wide linkage analysis of affected families, a candidate gene *RNF213* has been identified and subsequently knocked down in a zebrafish model.^104^ The phenotype generated by morpholino oligonucleotide injection was abnormal vessel sprouting and irregular vessel diameter, supporting the role of *RNF213* in vascular development and stability. In another study, a rare x-linked moyamoya syndrome was found to be caused by Xq28 deletions (removing *MTCP1/MTCP1NB* and *BRCC3*). In a functional study of *BRCC3*, morphant zebrafish were generated. Knockdown of this gene resulted in angiogenesis defects, which were also rescued by endothelium specific expression of *BRCC3*.^105^ These studies demonstrate the utility of zebrafish to serve in experiments of gene function, allowing for the visualization of abnormalities in the cranial vasculature *in vivo.* By establishing a model phenotype based on gene mutations found in humans, dissection of corresponding signal cascades can be performed. Establishment of these novel morphants and mutant lines also facilitates their integration into high-throughput screening platforms in search of small molecules that rescue the disease phenotype. Certain limitations of embryonic zebrafish in the study of arterial disease and vascular malformations must be acknowledged, and are centered on the apparent lack of pericytes and smooth muscle cells in the very young embryo, which are known to contribute to disease pathogenesis in humans.^106^

### Ischemic Stroke

Ischemic stroke, resulting from cerebral vascular occlusion, is a major cause of death and disability worldwide. With the exception of tissue plasminogen activator, there are no targeted medical therapies available, highlighting the need for accelerated drug discovery. The use of zebrafish in the study of ischemic stroke lags behind hemorrhagic stroke, and only relatively few preliminary research efforts have been published on the subject. A notable study describes the establishment of a zebrafish model of hypoxic–ischemic injury^107^, with a follow-up report from the same group describing the neuroprotective effects of a zinc chelator using the same model.^108^ Furthermore, certain hereditary forms of ischemic stroke in humans, such as cerebral autosomal dominant arteriopathy with subcortical infarcts and leukoencephalopathy have been modeled in the zebrafish.^109^ This model, generated by mutations in the *notch3* gene, has a phenotype in the fish typified by enlargement of vessels in the telencephalon and fin, disorganization of the normal stereotyped arrangement of vessels in the fin, and gaps in the arterial wall.

Even though limited work has been performed with ischemic stroke models, zebrafish represent a vast platform to investigate gene function. In particular, genes that control the expression of ion transport channels are central importance in understanding the pathophysiologic sequelae that follows an initial ischemic insult.^110^ For example, the cation-chloride co-transporter NKCC1 (sodium-potassium-chloride co-transporter 1) is one of the key ion channels that contributes to the development of cytotoxic and ionic edema following ischemia.^110, 111, 112, 113, 114^ This ion channel is well described in the zebrafish and has been shown to be important in the regulation of endolymph volume in the otic vesicle and swim bladder volume.^115^ Even though an inhibitor of this ion channel is available in humans (bumetanide), it is limited by low blood–brain barrier penetration^116^ and lack of specificity at high concentrations.^117^ The zebrafish represents an opportunity to discover another more selective NKCC1 inhibitor with better blood–brain barrier penetration, with potential as therapy to preempt post-ischemic cytotoxic and ionic edema. Several other channels implicated in dysregulation of the neurogliovascular unit following ischemic stroke, such as the *N*-methyl-D-aspartate receptor and acid-sensing ion channel, have been described in the zebrafish.^118, 119^

In addition to mitigating the secondary effects from ischemic stroke, such as hemorrhagic transformation and cerebral edema formation, an effort to understand the mechanisms of post-stroke recovery has the potential to uncover novel therapeutic targets. Specifically, a focus on adult neurogenesis and the migration of regenerating neurons in the post-injury recovery period is an ongoing area of research.^120, 121, 122^ These processes are being studied in the zebrafish, using adult fish as model organisms.^123, 124, 125^ Further refinements in experimental injury will allow for the study of brain recovery from injury, whether it is from stroke or trauma.

In any zebrafish model of cerebrovascular disease, it should be noted that many physiologic aspects of the cerebral circulation in zebrafish are not yet well known. Factors that have proven important in human ischemic stroke, such as collateral circulation^126^ and autoregulatory capacity^127^, are yet to be evaluated in the zebrafish.

## Summary

Zebrafish have been used as model organisms in the investigation of both hemorrhagic and ischemic stroke. They have been shown to be useful not only in the investigation of gene function, but also as a high-throughput drug discovery screening platform. With the conservation of many molecular mechanisms of disease among vertebrates, zebrafish experiments are poised to result in a better understanding and new therapeutics for human cerebrovascular disease.

## References

[bib1] Dickson M Gagnon JP Key factorsin the rising cost of new drug discovery and development Nat Rev Drug Discov 2004 3 417 429 15136789 10.1038/nrd1382

[bib2] Zon LI Peterson RT *In vivo* drug discovery in the zebrafish Nat Rev Drug Discov 2005 4 35 44 15688071 10.1038/nrd1606

[bib3] Kokel D Bryan J Laggner C White R Cheung CYJ Mateus R Rapid behavior-based identification of neuroactive small molecules in the zebrafish Nat Chem Biol 2010 6 231 237 20081854 10.1038/nchembio.307 PMC2834185

[bib4] Lieschke GJ Currie PD Animal models of human disease: zebrafish swim into view Nat Rev Genet 2007 8 353 367 17440532 10.1038/nrg2091

[bib5] Jin S-W Beis D Mitchell T Chen J-N Stainier DY Cellular and molecular analyses of vascular tube and lumen formation in zebrafish Development 2005 132 5199 5209 16251212 10.1242/dev.02087

[bib6] Schwerte T Fritsche R Understanding cardiovascular physiology in zebrafish and Xenopus larvae: the use of microtechniques Comp Biochem Physiol A Mol Integr Physiol 2003 135 131 145 12727550 10.1016/s1095-6433(03)00044-8

[bib7] Peterson RT Shaw SY Peterson TA Milan DJ Zhong TP Schreiber SL Chemical suppression of a genetic mutation in a zebrafish model of aortic coarctation Nat Biotechnol 2004 22 595 599 15097998 10.1038/nbt963

[bib8] Malone MH Sciaky N Stalheim L Hahn KM Linney E Johnson GL Laser-scanning velocimetry: a confocal microscopy method for quantitative measurement of cardiovascular performance in zebrafish embryos and larvae BMC Biotechnol 2007 7 40 17623073 10.1186/1472-6750-7-40 PMC1955438

[bib9] Yaniv K Isogai S Castranova D Dye L Hitomi J Weinstein BM Live imaging of lymphatic development in the zebrafish Nat Med 2006 12 711 716 16732279 10.1038/nm1427

[bib10] Lawson ND Weinstein BM *In Vivo* imaging of embryonic vascular development using transgenic zebrafish Dev Biol 2002 248 307 318 12167406 10.1006/dbio.2002.0711

[bib11] Sullivan-Brown J Bisher ME Burdine RD Embedding, serial sectioning and staining of zebrafish embryos using JB-4 resin Nat Protoc 2010 6 46 55 21212782 10.1038/nprot.2010.165 PMC3122109

[bib12] Barbazuk WB Korf I Kadavi C Heyen J Tate S Wun E The syntenic relationship of the zebrafish and human genomes Genome Res 2000 10 1351 1358 10984453 10.1101/gr.144700 PMC310919

[bib13] Fisher S Grice EA Vinton RM Bessling SL McCallion AS Conservation of RET regulatory function from human to zebrafish without sequence similarity Science 2006 312 276 279 16556802 10.1126/science.1124070

[bib14] Lam SH Wu YL Vega VB Miller LD Spitsbergen J Tong Y Conservation of gene expression signatures between zebrafish and human liver tumors and tumor progression Nat Biotechnol 2005 24 73 75 16327811 10.1038/nbt1169

[bib15] Milan DJ Peterson TA Ruskin JN Peterson RT MacRae CA Drugs that induce repolarization abnormalities cause bradycardia in zebrafish Circulation 2003 107 1355 1358 12642353 10.1161/01.cir.0000061912.88753.87

[bib16] Golling G Amsterdam A Sun Z Antonelli M Maldonado E Chen W Insertional mutagenesis in zebrafish rapidly identifies genes essential for early vertebrate development Nat Genet 2002 31 135 140 12006978 10.1038/ng896

[bib17] Nasevicius A Ekker SC Effective targeted gene ‘knockdown'in zebrafish Nat Genet 2000 26 216 220 11017081 10.1038/79951

[bib18] Childs S Chen J-N Garrity DM Fishman MC Patterning of angiogenesis in the zebrafish embryo Development 2002 129 973 982 11861480 10.1242/dev.129.4.973

[bib19] Sander JD Cade L Khayter C Reyon D Peterson RT Joung JK Targeted gene disruption in somatic zebrafish cells using engineered TALENs Nat Biotechnol 2011 29 697 21822241 10.1038/nbt.1934 PMC3154023

[bib20] Cade L Reyon D Hwang WY Tsai SQ Patel S Khayter C Highly efficient generation of heritable zebrafish gene mutations using homo-and heterodimeric TALENs Nucleic Acids Res 2012 40 8001 8010 22684503 10.1093/nar/gks518 PMC3439908

[bib21] Sander JD Dahlborg EJ Goodwin MJ Cade L Zhang F Cifuentes D Selection-free zinc-finger-nuclease engineering by context-dependent assembly (CoDA) Nat Methods 2010 8 67 69 21151135 10.1038/nmeth.1542 PMC3018472

[bib22] Foley JE Maeder ML Pearlberg J Joung JK Peterson RT Yeh J-RJ Targeted mutagenesis in zebrafish using customized zinc-finger nucleases Nat Protoc 2009 4 1855 1868 20010934 10.1038/nprot.2009.209 PMC2814337

[bib23] Hwang WY Fu Y Reyon D Maeder ML Tsai SQ Sander JD Efficient genome editing in zebrafish using a CRISPR-Cas system Nat Biotechnol 2013 31 227 229 23360964 10.1038/nbt.2501 PMC3686313

[bib24] MacRae CA Peterson RT Zebrafish-based small molecule discovery Chem Biol 2003 10 901 908 14583256 10.1016/j.chembiol.2003.10.003

[bib25] Knudsen KA Rosand J Karluk D Greenberg SM Clinical diagnosis of cerebral amyloid angiopathy: validation of the Boston criteria Neurology 2001 56 537 539 11222803 10.1212/wnl.56.4.537

[bib26] Vonsattel JPG Myers RH Tessa Hedley-Whyte E Ropper AH Bird ED Richardson EP Cerebral amyloid angiopathy without and with cerebral hemorrhages: a comparative histological study Ann Neurol 1991 30 637 649 1763890 10.1002/ana.410300503

[bib27] Zlokovic BV Neurovascular mechanisms of Alzheimer's neurodegeneration Trends Neurosci 2005 28 202 208 15808355 10.1016/j.tins.2005.02.001

[bib28] Donnini S Solito R Cetti E Corti F Giachetti A Carra S Aβ peptides accelerate the senescence of endothelial cells *in vitro* and *in vivo*, impairing angiogenesis FASEB J 2010 24 2385 2395 20207941 10.1096/fj.09-146456

[bib29] Wedderburn CJ van Beijnum J Bhattacharya JJ Counsell CE Papanastassiou V Ritchie V Outcome after interventional or conservative management of unruptured brain arteriovenous malformations: a prospective, population-based cohort study Lancet Neurol 2008 7 223 230 18243054 10.1016/S1474-4422(08)70026-7

[bib30] Augustin HG Reiss Y EphB receptors and ephrinB ligands: regulators of vascular assembly and homeostasis Cell Tissue Res 2003 314 25 31 12905065 10.1007/s00441-003-0770-9

[bib31] Swift MR Weinstein BM Arterial–venous specification during development Circ Res 2009 104 576 588 19286613 10.1161/CIRCRESAHA.108.188805

[bib32] Lawson ND Vogel AM Weinstein BM Sonic hedgehog and vascular endothelial growth factor act upstream of the notch pathway during arterial endothelial differentiation Dev Cell 2002 3 127 136 12110173 10.1016/s1534-5807(02)00198-3

[bib33] Walker EJ Su H Shen F Degos V Jun K Young WL Bevacizumab attenuates VEGF-induced angiogenesis and vascular malformations in the adult mouse brain Stroke 2012 43 1925 1930 22569934 10.1161/STROKEAHA.111.647982 PMC3404823

[bib34] Coultas L Chawengsaksophak K Rossant J Endothelial cells and VEGF in vascular development Nature 2005 438 937 945 16355211 10.1038/nature04479

[bib35] Carlson TR Yan Y Wu X Lam MT Tang GL Beverly LJ Endothelial expression of constitutively active Notch4 elicits reversible arteriovenous malformations in adult mice Proc Natl Acad Sci USA 2005 102 9884 9889 15994223 10.1073/pnas.0504391102 PMC1175015

[bib36] Carlson TR Yan Y Wu X Lam MT Tang GL Beverly LJ Endothelial expression of constitutively active Notch4 elicits reversible arteriovenous malformations in adult mice Proc Natl Acad Sci USA 2005 102 9884 9889 15994223 10.1073/pnas.0504391102 PMC1175015

[bib37] Goumans M-J Valdimarsdottir G Itoh S Rosendahl A Sideras P ten Dijke P Balancing the activation state of the endothelium via two distinct TGF-β type I receptors EMBO J 2002 21 1743 1753 11927558 10.1093/emboj/21.7.1743 PMC125949

[bib38] Hong CC Peterson QP Hong JY Peterson RT Artery/vein specification is governed by opposing phosphatidylinositol-3 kinase and MAP kinase/ERK signaling Curr Biol 2006 16 1366 1372 16824925 10.1016/j.cub.2006.05.046 PMC1930149

[bib39] Jain RK Molecular regulation of vessel maturation Nat Med 2003 9 685 693 12778167 10.1038/nm0603-685

[bib40] Herbert SP Huisken J Kim TN Feldman ME Houseman BT Wang RA Arterial-venous segregation by selective cell sprouting: an alternative mode of blood vessel formation Science 2009 326 294 19815777 10.1126/science.1178577 PMC2865998

[bib41] Codd PJ Mitha AP Ogilvy CS A recurrent cerebral arteriovenous malformation in an adult J Neurosurg 2008 109 486 491 18759581 10.3171/JNS/2008/109/9/0486

[bib42] Sure U Butz N Schlegel J Siegel AM Wakat JP Mennel HD Endothelial proliferation, neoangiogenesis, and potential *de novo* generation of cerebrovascular malformations J Neurosurg 2001 94 972 977 11409527 10.3171/jns.2001.94.6.0972

[bib43] Johnson D Berg J Baldwin M Gallione C Marondel I Yoon S-J Mutations in the activin receptor–like kinase 1 gene in hereditary haemorrhagic telangiectasia type 2 Nat Genet 1996 13 189 195 8640225 10.1038/ng0696-189

[bib44] Corti P Young S Chen CY Patrick MJ Rochon ER Pekkan K Interaction between alk1 and blood flow in the development of arteriovenous malformations Development 2011 138 1573 1582 21389051 10.1242/dev.060467 PMC3062425

[bib45] Roman BL Pham VN Lawson ND Kulik M Childs S Lekven AC Disruption of acvrl1 increases endothelial cell number in zebrafish cranial vessels Development 2002 129 3009 3019 12050147 10.1242/dev.129.12.3009

[bib46] Walcott BP Smith ER Scott RM Orbach DB Pial arteriovenous fistulae in pediatric patients: associated syndromes and treatment outcome J Neurointerv Surg 2013 5 10 14 22213836 10.1136/neurintsurg-2011-010168

[bib47] Eerola I Boon LM Mulliken JB Burrows PE Dompmartin A Watanabe S Capillary malformation–arteriovenous malformation, a new clinical and genetic disorder caused by RASA1 mutations Am J Hum Genet 2003 73 1240 1249 14639529 10.1086/379793 PMC1180390

[bib48] Kim H Marchuk DA Pawlikowska L Chen Y Su H Yang G Genetic considerations relevant to intracranial hemorrhage and brain arteriovenous malformationsIn: Liang-Fu Z, Guohua X, Xian-Cheng C, Richard FK, Feng-Ping H, Ya H *et al* (eds).. Cerebral Hemorrhage Springer: Austria 2008 199 206 10.1007/978-3-211-09469-3_38 PMC2640934 19066109

[bib49] Leblanc GG Golanov E Awad IA Young WL Biology of vascular malformations of the brain Stroke 2009 40 e694 e702 19834013 10.1161/STROKEAHA.109.563692 PMC2810509

[bib50] Nishida T Faughnan ME Krings T Chakinala M Gossage JR Young WL Brain arteriovenous malformations associated with hereditary hemorrhagic telangiectasia: gene–phenotype correlations Am J Med Genet A 2012 158 2829 2834 22991266 10.1002/ajmg.a.35622 PMC3610331

[bib51] Simon M Franke D Ludwig M Aliashkevich AF Köster G Oldenburg J Association of a polymorphism of the ACVRL1 gene with sporadic arteriovenous malformations of the central nervous system J Neurosurg 2006 104 945 949 16776339 10.3171/jns.2006.104.6.945

[bib52] Sturiale CL Puca A Sebastiani P Gatto I Albanese A Di Rocco C Single nucleotide polymorphisms associated with sporadic brain arteriovenous malformations: where do we stand Brain 2013 136 665 681 22975391 10.1093/brain/aws180

[bib53] Siekmann R Wakhloo AK Lieber BB Gounis MJ Divani AA Hopkins LN Modification of a previously described arteriovenous malformation model in the swine: endovascular and combined surgical/endovascular construction and hemodynamics Am J Neuroradiol 2000 21 1722 1725 11039356 PMC8174859

[bib54] Hao Q Zhu Y Su H Shen F Yang G-Y Kim H VEGF induces more severe cerebrovascular dysplasia in Eng+/− than in Alk1+/− mice Transl Stroke Res 2010 1 197 201 20640035 10.1007/s12975-010-0020-x PMC2902730

[bib55] Hao Q Su H Marchuk DA Rola R Wang Y Liu W Increased tissue perfusion promotes capillary dysplasia in the ALK1-deficient mouse brain following VEGF stimulation Am J Physiol-Heart Circ Physiol 2008 295 H2250 H2256 18835925 10.1152/ajpheart.00083.2008 PMC2614529

[bib56] Walker EJ Su H Shen F Choi EJ Oh SP Chen G Arteriovenous malformation in the adult mouse brain resembling the human disease Ann Neurol 2011 69 954 962 21437931 10.1002/ana.22348 PMC3117949

[bib57] Walcott BP Smith ER Scott RM Orbach DB Dural arteriovenous fistulae in pediatric patients: associated conditions and treatment outcomes J Neurointerv Surg 2013 5 6 9 22213835 10.1136/neurintsurg-2011-010169

[bib58] Lasjaunias P Chiu M Ter Brugge K Tolia A Hurth M Bernstein M Neurological manifestations of intracranial dural arteriovenous malformations J Neurosurg 1986 64 724 730 3701421 10.3171/jns.1986.64.5.0724

[bib59] A Randomized Trial of Unruptured Brain Arteriovenous Malformations (ARUBA). Available at http://www.ninds.nih.gov/news_and_events/news_articles/ARUBA_trial_results.htm . 10.1007/978-3-211-76589-0_1 18496936

[bib60] Wiebers DO Unruptured intracranial aneurysms: natural history, clinical outcome, and risks of surgical and endovascular treatment Lancet 2003 362 103 110 12867109 10.1016/s0140-6736(03)13860-3

[bib61] Molyneux AJ Kerr RS Yu L-M Clarke M Sneade M Yarnold JA International subarachnoid aneurysm trial (ISAT) of neurosurgical clipping versus endovascular coiling in 2143 patients with ruptured intracranial aneurysms: a randomised comparison of effects on survival, dependency, seizures, rebleeding, subgroups, and aneurysm occlusion Lancet 2005 366 809 817 16139655 10.1016/S0140-6736(05)67214-5

[bib62] Taylor CL Yuan Z Selman WR Ratcheson RA Rimm AA Cerebral arterial aneurysm formation and rupture in 20,767 elderly patients: hypertension and other risk factors J Neurosurg 1995 83 812 819 7472548 10.3171/jns.1995.83.5.0812

[bib63] Juvela S Porras M Poussa K Natural history of unruptured intracranial aneurysms: probability of and risk factors for aneurysm rupture J Neurosurg 2000 93 379 387 10969934 10.3171/jns.2000.93.3.0379

[bib64] Foroud T Sauerbeck L Brown R Anderson C Woo D Kleindorfer D Genome screen to detect linkage to intracranial aneurysm susceptibility genes: the Familial Intracranial Aneurysm (FIA) study Stroke 2008 39 1434 1440 18323491 10.1161/STROKEAHA.107.502930 PMC2435164

[bib65] Ozturk AK Nahed BV Bydon M Bilguvar K Goksu E Bademci G Molecular genetic analysis of two large kindreds with intracranial aneurysms demonstrates linkage to 11q24-25 and 14q23-31 Stroke 2006 37 1021 1027 16497978 10.1161/01.STR.0000206153.92675.b9

[bib66] Nahed BV Seker A Guclu B Ozturk AK Finberg K Hawkins AA Mapping a Mendelian form of intracranial aneurysm to 1p34.3-p36.13 Am J Hum Genet 2005 76 172 179 15540160 10.1086/426953 PMC1196421

[bib67] Nahed BV Bydon M Ozturk AK Bilguvar K Bayrakli F Gunel M Genetics of intracranial aneurysms Neurosurgery 2007 60 213 225discussion 225-6. 17290171 10.1227/01.NEU.0000249270.18698.BB

[bib68] Mangos S Lam P-y Zhao A Liu Y Mudumana S Vasilyev A The ADPKD genes pkd1a/b and pkd2 regulate extracellular matrix formation Dis Model Mech 2010 3 354 365 20335443 10.1242/dmm.003194 PMC2860853

[bib69] Hoh BL Rabinov JD Pryor JC Ogilvy CS A modified technique for using elastase to create saccular aneurysms in animals that histologically and hemodynamically resemble aneurysms in human Acta Neurochir 2004 146 705 711 15197614 10.1007/s00701-004-0276-6

[bib70] Cloft HJ Altes TA Marx WF Raible RJ Hudson SB Helm GA Endovascular creation of an *in vivo* bifurcation aneurysm model in rabbits Radiology 1999 213 223 228 10540666 10.1148/radiology.213.1.r99oc15223

[bib71] Park SO Wankhede M Lee YJ Choi EJ Fliess N Choe SW Real-time imaging of *de novo* arteriovenous malformation in a mouse model of hereditary hemorrhagic telangiectasia J Clin Invest 2009 119 3487 3496 19805914 10.1172/JCI39482 PMC2769195

[bib72] Nuki Y Tsou TL Kurihara C Kanematsu M Kanematsu Y Hashimoto T Elastase-induced intracranial aneurysms in hypertensive mice Hypertension 2009 54 1337 1344 19884566 10.1161/HYPERTENSIONAHA.109.138297 PMC2797444

[bib73] Bouzeghrane F Naggara O Kallmes DF Berenstein A Raymond J *In vivo* experimental intracranial aneurysm models: a systematic review AJNR. Am J Neuroradiol 2010 31 418 423 19875466 10.3174/ajnr.A1853 PMC7963965

[bib74] Schmitt CE Holland MB Jin SW Visualizing vascular networks in zebrafish: an introduction to microangiography Methods Mol Biol 2012 843 59 67 22222521 10.1007/978-1-61779-523-7_6

[bib75] Kamei M Isogai S Pan W Weinstein BM Imaging blood vessels in the zebrafish Methods Cell Biol 2010 100 27 54 21111213 10.1016/B978-0-12-384892-5.00002-5

[bib76] Labauge P Denier C Bergametti F Tournier-Lasserve E Genetics of cavernous angiomas Lancet Neurol 2007 6 237 244 17303530 10.1016/S1474-4422(07)70053-4

[bib77] Aiba T Tanaka R Koike T Kameyama S Takeda N Komata T Natural history of intracranial cavernous malformations J Neurosurg 1995 83 56 59 7782850 10.3171/jns.1995.83.1.0056

[bib78] Kondziolka D Lunsford LD Kestle JR The natural history of cerebral cavernous malformations J Neurosurg 1995 83 820 824 7472549 10.3171/jns.1995.83.5.0820

[bib79] Asaad WF Walcott BP Nahed BV Ogilvy CS Operative management of brainstem cavernous malformations Neurosurg Focus 2010 29 E10 20809751 10.3171/2010.6.FOCUS10134

[bib80] Churl-Su K Sameer AS Brian PW Jonathan N Emad NE Christopher SO Long-term seizure outcomes following resection of supratentorial cavernous malformations Clin Neurol Neurosurg 2013 115 2377 2381 24075713 10.1016/j.clineuro.2013.08.024

[bib81] Amin-Hanjani S Ogilvy CS Candia GJ Lyons S Chapman PH Stereotactic radiosurgery for cavernous malformations: Kjellberg's experience with proton beam therapy in 98 cases at the Harvard Cyclotron Neurosurgery 1998 42 1229 1236 9632180 10.1097/00006123-199806000-00013

[bib82] Pollock BE Garces YI Stafford SL Foote RL Schomberg PJ Link MJ Stereotactic radiosurgery of cavernous malformations J Neurosurg 2000 93 987 991 11117872 10.3171/jns.2000.93.6.0987

[bib83] Kondziolka D Lunsford LD Flickinger JC Kestle JR Reduction of hemorrhage risk after stereotactic radiosurgery for cavernous malformations J Neurosurg 1995 83 825 831 7472550 10.3171/jns.1995.83.5.0825

[bib84] Zabramski JM Wascher TM Spetzler RF Johnson B Golfinos J Drayer BP The natural history of familial cavernous malformations: results of an ongoing study J Neurosurg 1994 80 422 432 8113854 10.3171/jns.1994.80.3.0422

[bib85] Laberge-le Couteulx S Jung HH Labauge P Houtteville J-P Lescoat C Cecillon M Truncating mutations in CCM1, encoding KRIT1, cause hereditary cavernous angiomas Nat Genet 1999 23 189 193 10508515 10.1038/13815

[bib86] Günel M Awad IA Finberg K Anson JA Steinberg GK Batjer HH A founder mutation as a cause of cerebral cavernous malformation in Hispanic Americans N Engl J Med 1996 334 946 951 8596595 10.1056/NEJM199604113341503

[bib87] Craig HD Günel M Cepeda O Johnson EW Ptacek L Steinberg GK Multilocus linkage identifies two new loci for a mendelian form of stroke, cerebral cavernous malformation, at 7p15–13 and 3q25. 2–27 Hum Mol Genet 1998 7 1851 1858 9811928 10.1093/hmg/7.12.1851

[bib88] Chen L Tanriover G Yano H Friedlander R Louvi A Gunel M Apoptotic functions of PDCD10/CCM3, the gene mutated in cerebral cavernous malformation 3 Stroke 2009 40 1474 1481 19246713 10.1161/STROKEAHA.108.527135 PMC2709460

[bib89] Gunel M Laurans MS Shin D DiLuna ML Voorhees J Choate K KRIT1, a gene mutated in cerebral cavernous malformation, encodes a microtubule-associated protein Proc Natl Acad Sci 2002 99 10677 10682 12140362 10.1073/pnas.122354499 PMC125011

[bib90] He Y Zhang H Yu L Gunel M Boggon TJ Chen H Stabilization of VEGFR2 signaling by cerebral cavernous malformation 3 is critical for vascular development Sci Signal 2010 3 ra26 20371769 10.1126/scisignal.2000722 PMC3052863

[bib91] Louvi A Chen L Zhang H Min W Günel M Loss of cerebral cavernous malformation 3 (Ccm3) in neuroglia leads to CCM and vascular pathology Proc Natl Acad Sci 2011 108 3737 3742 21321212 10.1073/pnas.1012617108 PMC3048113

[bib92] Faurobert E Albiges-Rizo C Recent insights into cerebral cavernous malformations: a complex jigsaw puzzle under construction FEBS J 2010 277 1084 1096 20096036 10.1111/j.1742-4658.2009.07537.x PMC3076058

[bib93] Stainier D Fouquet B Chen J-N Warren KS Weinstein BM Meiler SE Mutations affecting the formation and function of the cardiovascular system in the zebrafish embryo Development 1996 123 285 292 9007248 10.1242/dev.123.1.285

[bib94] Yoruk B Gillers BS Chi NC Scott IC Ccm3 functions in a manner distinct from Ccm1 and Ccm2 in a zebrafish model of CCM vascular disease Dev Biol 2012 362 121 131 22182521 10.1016/j.ydbio.2011.12.006

[bib95] Gore AV Lampugnani MG Dye L Dejana E Weinstein BM Combinatorial interaction between CCM pathway genes precipitates hemorrhagic stroke Dis Model Mech 2008 1 275 281 19093037 10.1242/dmm.000513 PMC2590810

[bib96] Mably JD Chuang LP Serluca FC Mohideen M-AP Chen J-N Fishman MC Santa and valentine pattern concentric growth of cardiac myocardium in the zebrafish Development 2006 133 3139 3146 16873582 10.1242/dev.02469

[bib97] Hogan BM Bussmann J Wolburg H Schulte-Merker S ccm1 cell autonomously regulates endothelial cellular morphogenesis and vascular tubulogenesis in zebrafish Hum Mol Genet 2008 17 2424 2432 18469344 10.1093/hmg/ddn142

[bib98] Scott RM Smith ER Moyamoya disease and moyamoya syndrome NEngl J Med 2009 360 1226 1237 10.1056/NEJMra0804622 19297575

[bib99] Scott RM Smith JL Robertson RL Madsen JR Soriano SG Rockoff MA Long-term outcome in children with moyamoya syndrome after cranial revascularization by pial synangiosis J Neurosurg 2004 100 142 149 14758941 10.3171/ped.2004.100.2.0142

[bib100] Ishikawa T Houkin K Kamiyama H Abe H Effects of surgical revascularization on outcome of patients with pediatric moyamoya disease Stroke 1997 28 1170 1173 9183345 10.1161/01.str.28.6.1170

[bib101] Matsushima T Inoue T Suzuki S Fujii K Fukui M Hasuo K Surgical treatment of moyamoya disease in pediatric patients-comparison between the results of indirect and direct revascularization procedures Neurosurgery 1992 31 401 405 1407421 10.1227/00006123-199209000-00003

[bib102] Ikeda H Sasaki T Yoshimoto T Fukui M Arinami T Mapping of a familial moyamoya disease gene to chromosome 3p24. 2-p26 Am J Hum Genet 1999 64 533 537 9973290 10.1086/302243 PMC1377762

[bib103] Sakurai K Horiuchi Y Ikeda H Ikezaki K Yoshimoto T Fukui M A novel susceptibility locus for moyamoya disease on chromosome 8q23 J Hum Genet 2004 49 278 281 15362573 10.1007/s10038-004-0143-6

[bib104] Liu W Morito D Takashima S Mineharu Y Kobayashi H Hitomi T Identification of RNF213 as a susceptibility gene for moyamoya disease and its possible role in vascular development PloS ONE 2011 6 e22542 21799892 10.1371/journal.pone.0022542 PMC3140517

[bib105] Miskinyte S Butler MG Hervé D Sarret C Nicolino M Petralia JD Loss of BRCC3 deubiquitinating enzyme leads to abnormal angiogenesis and is associated with syndromic moyamoya Am J Hum Genet 2011 88 718 728 21596366 10.1016/j.ajhg.2011.04.017 PMC3113251

[bib106] Santoro MM Pesce G Stainier DY Characterization of vascular mural cells during zebrafish development Mech Dev 2009 126 638 649 19539756 10.1016/j.mod.2009.06.1080 PMC2732398

[bib107] Yu X Li YV Zebrafish as an alternative model for hypoxic-ischemic brain damage Int J Physiol Pathophysiol Pharmacol 2011 3 88 21760967 PMC3134003

[bib108] Yu X Li YV Neuroprotective effect of zinc chelator DEDTC in a Zebrafish (Danio rerio) model of hypoxic brain injury Zebrafish 2013 10 30 35 23461417 10.1089/zeb.2012.0777

[bib109] Zaucker A Mercurio S Sternheim N Talbot WS Marlow FL Notch3 is essential for oligodendrocyte development and vascular integrity in zebrafish Dis Model Mech 2013 6 1246 1259 23720232 10.1242/dmm.012005 PMC3759344

[bib110] Kahle KT Simard JM Staley KJ Nahed BV Jones PS Sun D Molecular mechanisms of ischemic cerebral edema: role of electroneutral ion transport Physiology 2009 24 257 265 19675357 10.1152/physiol.00015.2009

[bib111] Chen H Luo J Kintner DB Shull GE Sun D Na+-dependent chloride transporter (NKCC1)-null mice exhibit less gray and white matter damage after focal cerebral ischemia J Cereb Blood Flow Metab 2005 25 54 66 15678112 10.1038/sj.jcbfm.9600006

[bib112] Su G Kintner DB Flagella M Shull GE Sun D Astrocytes from Na+-K+-Cl−cotransporter-null mice exhibit absence of swelling and decrease in EAA release Am J Physiol Cell Physiol 2002 282 C1147 C1160 11940530 10.1152/ajpcell.00538.2001

[bib113] Walcott BP Kahle KT Simard JM Novel treatment targets for cerebral edema Neurotherapeutics 2012 9 65 72 22125096 10.1007/s13311-011-0087-4 PMC3271162

[bib114] Kahle KT Gerzanich V Simard JM Molecular mechanisms of microvascular failure in CNS injury–synergistic roles of NKCC1 and SUR1/TRPM4 J Neurosurg 2010 113 622 20035575 10.3171/2009.11.JNS081052 PMC3032917

[bib115] Abbas L Whitfield TT Nkcc1 (Slc12a2) is required for the regulation of endolymph volume in the otic vesicle and swim bladder volume in the zebrafish larva Development 2009 136 2837 2848 19633174 10.1242/dev.034215 PMC2730410

[bib116] Li Y Cleary R Kellogg M Soul JS Berry GT Jensen FE Sensitive isotope dilution liquid chromatography/tandem mass spectrometry method for quantitative analysis of bumetanide in serum and brain tissue J Chromatogr B Analyt Technol Biomed Life sci 2011 879 998 1002 10.1016/j.jchromb.2011.02.018 PMC3727383 21414852

[bib117] Payne JA Functional characterization of the neuronal-specific K-Cl cotransporter: implications for [K+]o regulation Am J Physiol 1997 273 (5 Pt 1 C1516 C1525 9374636 10.1152/ajpcell.1997.273.5.C1516

[bib118] Paukert M Sidi S Russell C Siba M Wilson SW Nicolson T A family of acid-sensing Ion channels from the Zebrafish widespread expression in the central nervous system suggests a conserved role in neuronal communication J Biol Chem 2004 279 18783 18791 14970195 10.1074/jbc.M401477200

[bib119] Chen J Patel R Friedman TC Jones KS The behavioral and pharmacological actions of NMDA receptor antagonism are conserved in zebrafish larvae Int J Comp Psychol 2010 23 82 21278812 PMC3027073

[bib120] Tsai PT Ohab JJ Kertesz N Groszer M Matter C Gao J A critical role of erythropoietin receptor in neurogenesis and post-stroke recovery J Neurosci 2006 26 1269 1274 16436614 10.1523/JNEUROSCI.4480-05.2006 PMC6674578

[bib121] Ohab JJ Fleming S Blesch A Carmichael ST A neurovascular niche for neurogenesis after stroke J Neurosci 2006 26 13007 13016 17167090 10.1523/JNEUROSCI.4323-06.2006 PMC6674957

[bib122] Sanai N Tramontin AD Quiñones-Hinojosa A Barbaro NM Gupta N Kunwar S Unique astrocyte ribbon in adult human brain contains neural stem cells but lacks chain migration Nature 2004 427 740 744 14973487 10.1038/nature02301

[bib123] Adolf B Chapouton P Lam CS Topp S Tannhäuser B Strähle U Conserved and acquired features of adult neurogenesis in the zebrafish telencephalon Dev Biol 2006 295 278 293 16828638 10.1016/j.ydbio.2006.03.023

[bib124] Grandel H Kaslin J Ganz J Wenzel I Brand M Neural stem cells and neurogenesis in the adult zebrafish brain: origin, proliferation dynamics, migration and cell fate Dev Biol 2006 295 263 277 16682018 10.1016/j.ydbio.2006.03.040

[bib125] Kizil C Kaslin J Kroehne V Brand M Adult neurogenesis and brain regeneration in zebrafish Dev Neurobiol 2012 72 429 461 21595047 10.1002/dneu.20918

[bib126] Liebeskind DS Collateral circulation Stroke 2003 34 2279 2284 12881609 10.1161/01.STR.0000086465.41263.06

[bib127] Aaslid R Lindegaard K-F Sorteberg W Nornes H Cerebral autoregulation dynamics in humans Stroke 1989 20 45 52 2492126 10.1161/01.str.20.1.45

